# CDC42 Use in Viral Cell Entry Processes by RNA Viruses

**DOI:** 10.3390/v7122955

**Published:** 2015-12-10

**Authors:** Thomas Swaine, Matthias T. Dittmar

**Affiliations:** Queen Mary University of London, Barts and The London School of Medicine and Dentistry, Blizard Institute, 4 Newark Street, London E1 2AT, UK; ha09054@qmul.ac.uk

**Keywords:** Cdc42, Rho GTPase, HIV-1, cell entry, RNA virus, RSV, Ebola virus, coronavirus, rotavirus

## Abstract

The cellular actin cytoskeleton presents a barrier that must be overcome by many viruses, and it has become increasingly apparent many viral species have developed a diverse repertoire of mechanisms to hijack cellular actin-regulating signalling pathways as part of their cell entry processes. The Rho family GTPase Cdc42 is appreciated as a key moderator of cellular actin dynamics, and the development of specific Cdc42-inhibiting agents has given us an unprecedented ability to investigate its individual role in signalling pathways. However, investigative use of said agents, and the subsequent characterisation of the role Cdc42 plays in viral entry processes has been lacking. Here, we describe the current literature on the role of Cdc42 in human immunodeficiency virus (HIV)-1 cell entry, which represents the most investigated instance of Cdc42 function in viral cell entry processes, and also review evidence of Cdc42 use in other RNA virus cell entries, demonstrating prime areas for more extensive research using similar techniques.

## 1. Introduction

The cellular actin cytoskeleton is not a static phenomenon solely providing structural integrity to cells, and is instead constitutionally active throughout cellular life and involved in key processes such as intracellular organisation, motility, and intracellular transport within and out of cells [1,2,3]. As such, it is evident that viral entry into cells goes beyond the initial binding to virus-specific cell surface receptors, and in fact necessitates interaction with the actin cytoskeleton and its regulators that would otherwise act as barriers to effective infection [4]. Given the unparalleled diversity demonstrated by virus species, it is unsurprising that many viruses have evolved unique methods of not only subverting the barrier cellular actin presents, but even hijacking its chief regulators, the Rho family GTPases [5], to actively promote viral entry and subsequent nuclear infiltration.

**Figure 1:**
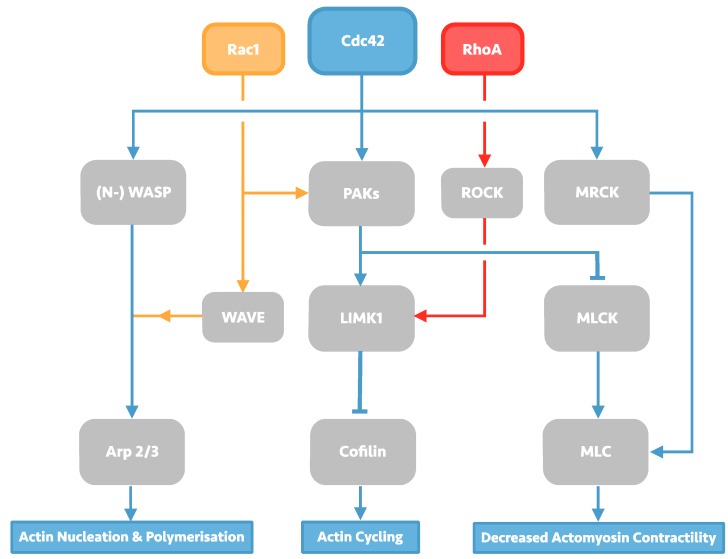
**Overview of Rho GTPase signalling, with a focus on Cdc42 signalling.** Cdc42 signalling can affect changes in actin dynamics through its three downstream effectors: the actin-polymerising protein Arp2/3, Cofilin, and myosin light chain (MLC). Arp 2/3 activation takes place through activation of Wiskott-Alrdich syndrome (WASP) scaffolding proteins in Cdc42 signalling, but can also be activated by Rac1 via WASP-family verprolin-homologous protein (WAVE) proteins. Activated WASP/WAVE proteins induce Arp2/3-led actin nucleation and polymerisation, producing actin meshwork. Cofilin activity is induced by protein activated kinases (PAKs) signalling, which may also be induced by Rac1 signalling. Activated PAKs can then activate LIM domain kinase 1 (LIMK1), leading to subsequent Cofilin phosphorylation and inhibition of its actin-severing function; LIMK1 may also be activated by RhoA via the serine/threonine kinase ROCK. PAKs signalling can also cause decreased actomyosin contractility by phosphorylating myosin light chain kinase (MLCK), allowing myosin light chain (MLC) activation. Cdc42 is also capable of activating MLC directly through myotonic dystrophy kinase-related Cdc42-binding kinases (MRCK).

The Rho family GTPases constitute a diverse group of cell signalling molecules present in all eukaryotic organisms, and play an integral role in the control of cellular actin dynamics, cumulating in macro-effects on cell morphology, membrane trafficking, and adhesions. Of the 23 related proteins which form the family, 22 are expressed in mammals, of which the Rac1 isoform, RhoA isoform, and Cdc42 have been subject to the most study and characterisation. RhoA, Rac1, and Cdc42 have been long known to produce individual characteristic effects on the cellular-cytoskeleton including lamellipodia, filopodia and stress fibre formation [6]. The individual effects of each of these Rho GTPases are enacted by the downstream effectors they regulate, which include the Arp2/3 complex, Wiskott-Alrdich syndrome (WASP) proteins, and myosin light chain kinase and phosphatase (MLCK), resulting in a diverse portfolio of effects on actin-dynamics that is summarised in Figure 1.

Key to the regulatory actions of Rho GTPases is their capacity to cycle between active and inactive states. Most Rho GTPases bind to both GTP and GDP, are capable of exerting intrinsic GTPase activity. When bound to GTP, Rho GTPases are in an active state and are able to bind and activate downstream signalling molecules, realising their effects on the cellular actin-network. Cycling between the active GTP-bound and inactive GDP-bound state can spontaneously occur in Rho GTPases due to their intrinsic GTPase activity, but within cells is regulated by three classes of related molecules: guanine exchange factors (GEFs), GTPase activating proteins (GAPs), and guanine nucleotide-dissociation inhibitors (GDIs).

**Figure 2:**
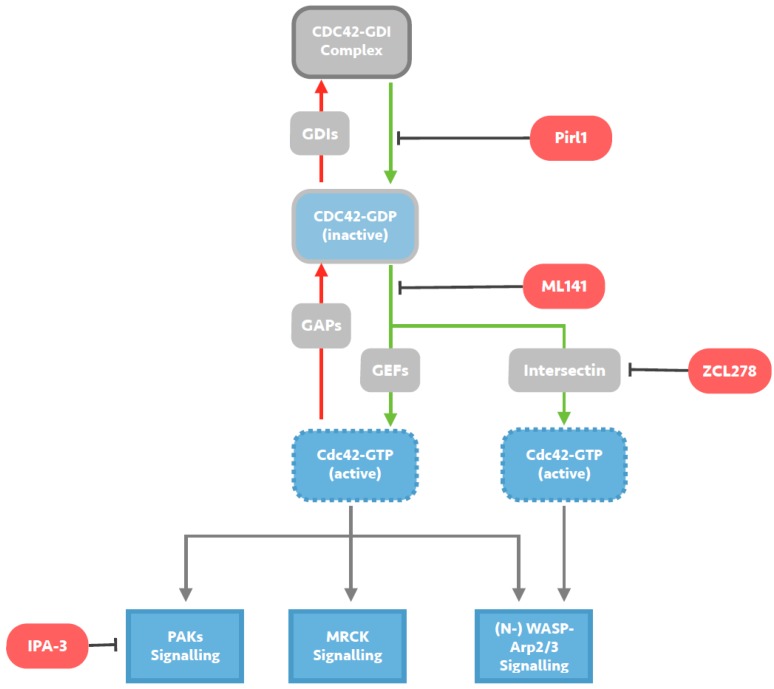
**Mechanisms of small molecule inhibition of Cdc42 signalling.** Cdc42 activity is regulated by cycling from a GTP-bound active state to a GDP-bound inactive state. Guanine exchange factors (GEFs) such as Intersectin, promote the exchange of GDP for GTP and activate Cdc42, whilst GTPase activating proteins (GAPs) catalyse the intrinsic GTPase function and inactivate Cdc42. Guanine nucleotide-dissociation inhibitors (GDIs) sequester GDP-bound Cdc42, maintaining a pool of inactive Cdc42. Pirl1 is a small molecule inhibitor of actin assembly thought to act by inhibiting activation of Cdc42/GDI complexes, precluding guanine nucleotide exchange on Cdc42 and any subsequent interaction with downstream effectors. ML141 is a selective reversible non-competitive allosteric inhibitor of Cd42, preventing GTP binding to the active site of Cdc42 without targeting any Cdc42-regulating molecules. ZCL278 acts by selectively blocking interactions between Cdc42 and the Cdc42-specific GEF Intersectin, likely predominantly preventing (N-) WASP-Arp2/3 signalling. IPA-3 is a small molecule inhibitor of downstream Cdc42 signalling, selectively inhibiting group 1 PAKs by targeting the auto-regulatory mechanism present in this group.

Although each Rho GTPase is associated with a characteristic effect on the cellular cytoskeleton, there is a great degree of overlap, crosstalk and dynamic action within Rho GTPase signalling pathways [7,8]. This can be demonstrated by considering the LIM domain kinase (LIMK)s-cofilin pathway, which not only unites all three major Rho GTPases, but is also capable of both F-actin polymerisation and de-polymerisation effects. Coupled with small and globular morphologies exposing few targetable sites for inhibitors, the characterisation of specific Rho GTPase signalling pathways has presented an extremely challenging task, and attempts to study individual GTPase function has been heavily restricted in the past, with initial attempts relying on the use of indiscriminate agents like Jasplakinolide [9] and Clostridium Toxin B [10], either target the entire cellular cytoskeleton or all its GTPase regulators. However, owing to the importance Rho GTPases appear to play in malignancy [11,12,13,14], progress in recent years has resulted in the development of several specific RhoA-, Rac1-, and Cdc42-inhibitors (illustrated in Figure 2), affording us an unprecedented capacity to study Rho GTPase involvement in viral processes. Despite these advancements, adoption of novel GTPase inhibitors has been extremely limited in this field, and even in the study of human immunodeficiency virus (HIV)-1 cell entry, where application of these agents has been most prolific, investigation has been unwarrantedly skewed towards targeting RhoA and Rac1, with the role of Cdc42 left mostly overlooked. To correct these oversights, this review aims to first present the growing evidence base for a primary role for Cdc42 signalling in HIV-1 cell entry arrived upon using novel specific inhibitory agents, and then to highlight a number of other RNA virus species in which the literature suggests similar study is likely to achieve similar success.

## 2. Investigation of the HIV-1 Entry Process

HIV-1 selectively infects human CD4+ cells, which include T-cells and macrophage lineage cells. Cell entry involves usage of both the CD4 cell surface receptor, and one of two chemokine co-receptors depending on viral tropism, CXCR4 or CCR5, to facilitate membrane fusion and viral core release into the cytoplasm. This fusogenic entry process necessitates interaction with the cellular actin cytoskeleton, however given the diversity of CD4+ cells and their subsequent actin dynamics, a universal blueprint for HIV-1-actin entry interactions in CD4+ cells would not be expected. Indeed, mature macrophages and dendritic cells exhibit a constitutively active cytoskeleton with constant endocytic and actin remodelling activity as part of their intrinsic function, whilst this is not entirely mirrored in the principle targets of HIV-1 disease: primary CD4+ T-cells. 

Primary CD4+ T-cells exhibit a dichotomy of actin dynamics [15]: in activated T-cells there is a polarized migratory morphology brought about by an active and plastic cytoskeleton; in rounded resting T-cells, which include na**ï**ve and memory subsets, a far more restricted cytoskeletal activity is observed, alongside a relative resistance to infection [16,17,18,19,20]. Although infection and subsequent depletion of activated T-cells is a leading process in the natural morbidity and mortality of HIV-1 infection, it is infection of resting T-cells that constitutes the principle barrier to cure. Activation of resting T-cells allows reestablishment of viremia when treatment is interrupted [21,22], therefore understanding viral entry into resting T-cells is of paramount interest and relevance.

Entry into these resting T-cells is considered a fusogenic process, yet the static cortical actin found in this subset should constitute a barrier to successful infection. This was initially evidenced by Yu *et al.* [23], who demonstrated that HIV-1 pseudo-typed with vesicular stomatitis virus glycoprotein (VSV-G) was incapable of infecting resting T-cells through its obligate endocytotic process. In contrast, wild-type X-4 tropic HIV-1 was found to be capable of doing so, suggesting that the HIV gp120 receptor binding, and subsequent chemokine signalling, induce necessary changes in target cell actin dynamics as part of the HIV-1 cell entry process.

Accepting this key need for viral induction of cellular actin disruption in the HIV-1 entry process of primary T-cells, various studies have investigated and aimed to characterize the underlying signalling pathways involved [24,25,26,27,28,29], resulting in the production of a synthesised model by Spear *et al.* in 2012 [30]. In this review, Spear *et al.* propose a multi-staged HIV-1 CXCR4-induced Rac1-protein activated kinases (PAK) signalling pathway as the principle agent in overcoming the cortical F-actin barrier. In the first stage, HIV-1 gp120 glycoprotein binding with CD4 and CXCR4 during the entry process leads to Rac-PAK-LIMK1 signalling causing cofilin phosphorylation and actin polymerisation, which is thought to prevent receptor internalisation and allow stabilisation of the HIV-receptor aggregates for subsequent fusion. Following fusion and viral entry, Gi alpha subunit (Gαi) signalling by activated CXCR4 causes compensatory de-phosphorylation of cofilin disrupting the cortical actin and allowing intracellular migration of HIV-1. Finally, the intracellular migration of HIV-1 was thought to take place via the process of actin-treadmilling driven by the de-phosphorylated cofilin. Later work would further characterise this as being an Arp2/3-dependent process [31]; experimental inhibition of Arp2/3 activity using Arp2/3 knockdown and the inhibitory agent CK548 was found to result in restricted HIV-1 nuclear migration, suggesting thatGP120-co-receptor signalling cumulates in Arp2/3 activity.

Though the initial model by Spear *et al.* [30] presented Rac1 as the principal GTPase in this process, much of the work drawn on in their review in fact implicated involvement from the other GTPases and effector molecules, including RhoA, ROCK, Cdc42, and Arp 2/3. Indeed, subsequent attempts to specifically inhibit Rac1 activity have since provided results discrediting a leading role for this particular GTPase. Pontow *et al*. [32] employed NSC23766, a specific inhibitor of the principal Rac1 guanine nucleotide exchange factors (GEF), Tiam-1, and demonstrated that NSC23766 had an inhibitory effect on HIV-1 entry into CD4 and chemokine receptor expressing glioma cells. However attempts to further this finding proved unsuccessful [33], and use of NSC23766 and the other Rac1 inhibiting agents EHT1864 and ITX3 were unable to inhibit HIV-1 infectivity in the PM1 cell line, a closer proxy for human T-cells [34]. As such, the later work from Spear *et al.* features an updated model that simply allows for viral induction of any of Rac1, RhoA, and Cdc42 signalling upon gp120 binding [31].

### Investigating a Specific Role for CDC42 in HIV entry

Whilst the current Cofilin/Arp2/3 signalling model proposes a shared importance for all major Rho GTPases in HIV-1 cell entry, this is unlikely to be a sufficiently detailed picture, and is instead reflective of the overlapping activities of the Rho GTPases and ongoing difficulties in targeting each specifically. Investigation has often relied on targeting downstream effectors such as protein activated kinases (PAKs) and Arp2/3 that are non-specific for individual GTPases, or through the use of complex techniques such as small interfering RNAs (siRNAs) and transfection with dominant-negative GTPases. However, the development of novel specific GTPase-inhibiting agents has facilitated some attempts to characterise the individual importance RhoA, Rac1 and Cdc42 play in the HIV-1 entry process. As previously discussed, though the Rac1 downstream effector Arp2/3 is strongly implicated, targeted inhibition of Rac1 signalling has not been shown to effectively inhibit T-cell entry by HIV-1 [34,35]. Considering the overlapping nature of Rac1 and Cdc42 signalling, these findings may support an alternative model in which the counterpart Cdc42-WASP-Arp2/3 signalling pathway instead plays the lead in the HIV-1 entry process.

Following this hypothesis, the possibility of a Cdc42-lead signalling pathway was investigated by our group in 2014 [35]. Two novel Cd42 inhibitors, ML141 [36,37,38] and ZCL278 [39], were employed to investigate inhibition of infection of three different HIV-1 pseudo-type virus strains [40,41]. The intersectin-specific ZCL278 was found to significantly inhibit the infectivity of all three pseudo-type viruses in a dose-dependent manner in both cell lines investigated (TZM-bl and PM1). At the highest doses used of 50 µM, the infectivity of X4 and R5 tropic pseudo-type viruses was reduced to 29.64% (X4) and 30.43% (R5) in TZM-bl cells, and to 22.96% for X4-tropic viruses in PM1 cells. The broader-acting ML141 showed almost no inhibitory effect on pseudo-type virus infectivity, and in fact increased the infectivity of X4-tropic virus in both TZM-bl and PM1 cells to a maximum effect of ~175% of control (12.5 µM ML141 on PM1 cells producing 172.66% infectivity).

Whilst ZCL278 is very specific, as an allosteric inhibitor of the intersectin-CDC42 binding, ML141 has blanket inhibitory effect on all Cdc42 activity. This indiscriminate inhibition of Cdc42 signalling may have had enigmatic effects on other Rho signalling pathways, and it was noted in the original characterization of ML141 that the agent had potential off-target effects on Rac1-regulating molecules [37,42]. Intersectin is specifically linked to the N-WASP-Arp2/3 pathway [43,44,45,46], logically suggesting that ZCL278 predominantly inhibits Cdc42-N-WASP-Arp2/3 signalling. In addition, because WASP/WAVE signalling exist as parallel pathways, and, as discussed earlier, activity of the shared WASP/WAVE downstream effector Arp2/3 has been previously implicated as necessary to HIV-1 nuclear migration [31], our data support the hypothesis that the Cdc42-N-WASP-Arp2/3 pathway, rather than Rac1-WAVE-Arp2/3, is key to HIV-1 T-cell entry process.

## 3. Cdc42 Involvement in other RNA Virus Cell Entry

Despite receiving less investigation than HIV-1, many other RNA virus species have been noted in recent years to employ analogous Cdc42 hijacking tactics in their target cell entry processes. As an illustrative—but not exhaustive—selection of these RNA species, the use of Cdc42 in the cell entry of Respiratory Syncytial Virus (RSV), Ebola virus (EBOV), and various Rotavirus (RV) and Coronavirus (CoV) strains are discussed in this review.

### 3.1. Respiratory Syncytial Virus

Similarly to other members of the paramyxovirus family, RSV was originally believed to enter target cells in a fusogenic manner via the RSV fusion (RSV-F) glycoprotein found in the viral envelope. This theory was evidenced by initial dequenching assays, in which fluorescent lipid probes in target cell plasma membranes dequenched when incubated in RSV virions, indicating a mixing and dilution of the target cell plasma membranes that was attributed to RSV fusion [47]. It was then demonstrated in several studies that the RSV entry process is independent of endosomal acidification [48,49,50], supporting fusogenic entry.

However, evidence in recent years suggests that the entry process may involve endocytotic processes too. Targeted siRNA screening in HeLa cells by Kolokolstov *et al.* [51] demonstrated that knockdown of various genes implicated in endocytosis function conferred a protection to RSV infection. Amongst the protective genes were clathrin light and heavy chain genes, which the author took to suggest a clathrin-mediated endocytotic entry process. Additionally PAK1 and intersectins 1 and 2—key components of Cdc42 actin signalling—were demonstrated to convey protection to RSV infection when inhibited. This was developed by San-Juan-Vergara *et al.* [52], who demonstrated using dual-wavelength imaging that RSV entry involves initial docking and hemifusion at cholesterol rich membrane domains. The group then identified that this is followed by a PAK1-dependent actin reorganisation, as the application of IPA-3, a small molecule PAK1 inhibitor, reduced RSV infectivity to about half that of untreated cells, again indicating a crucial role for Cdc42 signalling.

Most recently, a theory of macropinocytotic entry has been proposed by Krzyzaniak *et al.* [53], that clearly demonstrates a primary role for Cdc42 in RSV cell-entry. After demonstrating through F- and N-viral protein staining that RSV is endocytosed, and that this process is clathrin-, dynamin- and pH-independent, the group investigated the effect of various actin-disrupting agents on RSV infection. Though RhoA and Rac1 inhibition had a moderate effect, Cdc42 inhibition using PIRL1—a Cdc42/GDI complex activation inhibitor [54]—produced a severe restriction of RSV infectivity. In addition, inhibition of downstream Cdc42 effectors PAK1, WASP and ARP2/3 showed moderate decreases in infectivity when targeted individually.

### 3.2. Rotaviruses

Individual RV strains are thought to enter target cells through distinct endocytotic pathways that have been poorly characterised in the past, but are becoming increasingly well understood. Rhesus rotavirus (RRV) is one of the more extensively studied RV strains, and early attempts to determine its entry process using lysosomotropic agents and endosomal trafficking blockers [55,56,57] demonstrated that the entry process did not follow the classical endocytotic pathway. By using newer dominant negative protein transfection techniques Sánchez-San Martín *et al.* [58] were able to further characterise RRV entry as a dynamin-dependent and clathrin-, caveolae-independent process, later also described to be Cdc42-dependent [59]. Following on from this work, other strains of RRV (TFR-41, UK, and Wa) were characterised and utilise clathrin-dependent processes [60]. Marco *et al*. [61] later showed that expression of dominant negative variants of Cdc42 in target cells inhibits infection with the clathrin-dependent RV UK strain too, suggesting that despite differing substantially, all RV entry processes share a common Cdc42-dependence.

### 3.3. Coronaviruses

The entry routes for CoV are still debated, and various routes have been proposed for the various CoV strains. These include poorly characterised clathrin- and caveolae-independent entry pathways for severe acute respiratory syndrome (SARS) virus [62], lipid-raft and caveolae-mediated entry in human CoV strains [63], and classical clathrin endocytosis in the commonly studied mouse hepatitis coronavirus (MHV). A recent study using siRNA techniques has helped elucidated many aspects of MHV CoV entry, and also identified critical potential roles for Cdc42 in this process. Using a high throughput siRNA screen, Nomura *et al*. [63] demonstrated that silencing of two major constituents of the Arp2/3 complex resulted in significant reduction if MHV infectivity, and use of actin cytoskeleton altering agents in the early stages of infection had similar restrictive effects on infection, pointing to GTPase, and specifically Cdc42 involvement in the entry process. Furthermore, the authors noted that though they concluded MHV likely entered by clathrin endocytosis, infection was severely inhibited by ethylisopropyl amiloride (EIPA), an agent primarily associated with macropinocytosis blockage. EIPA is indeed known for its hallmark effect of inhibiting macropinocytosis, but actually causes this effect by preventing the necessary Rac1 and Cdc42 signalling involved [64]. These findings of restricted infectivity due to Arp2/3 knockdown, actin cytoskeleton disruption, and EIPA are strongly suggestive of Cdc42 signalling in MHV the cell entry process.

### 3.4. Ebola Virus

EBOV cell entry is known to be mediated by the fusion glycoprotein, and several theories for its entry process have been presented over time, with current opinion holding that entry can be either via clathrin-endocytosis or macropinocytosis [65]. Evidence suggesting Rho family GTPase involvement was first identified by Quinn *et al.* [66], who demonstrated RhoB and RhoC overexpression via plasmids resulted in increased susceptibility to EBOV infection in 293T cells. Later work investigating the macropinocytotic entry process builds on these findings, and indicates key roles for Cdc42; Mulherkar *et al.* [67] demonstrated that EBOV infectivity in macropinocytotic permissible cells was severely reduced by EIPA and actin polymerisation inhibitors. Furthermore, Pak-1 and the Rac1-related GTPase Arf6 were also investigated by the group, and it was determined that EBOV entry was dependent on Pak-1, but not Arf6, suggesting Cdc42 rather than Rac1 was the specific Rho GTPase involved. These findings are supported by Aleksandrowicz *et al.* [65], who reproduced the finding that EIPA inhibited EBOV infection. Despite contemporary global interest in the EBOV, further study specifically targeting Cdc42 has not been undertaken, leaving us unable to characterise this involvement further.

## 4. Discussion

The studies discussed here suggest that many RNA viruses share a dependence on Cdc42-signalling to achieve successful infection. In HIV-1 this involvement of Cdc42 has been most thoroughly characterised, likely because of its global burden and the ongoing exploration of the viral lifecycle warranted in the hope of identifying new potential drug targets. Whilst a spectrum of techniques ranging from the broader use of general actin inhibition, to more targeted investigations with siRNA knockdowns of specific signalling-related genes, have provided the bulk of initial data for HIV-1 Cdc42 use, it has only been through employing novel Cdc42-specific agents that the clearest indications of Cdc42 use have been elucidated. Without the use of such precise agents it is easy to overlook Cdc42 involvement and even misinterpret processes due to the oft-reported overlapping functions of Rho Family GTPases. Indeed, given that the study of various Cdc42-inhibitors in HIV-1 seems to have demonstrated that inhibition of all Cdc42 function may result in dysregulation of other Rho family GTPases, it is possible that the broadly-acting agents used in the past to “screen” for Cdc42 involvement in virus species’ entry processes may have produced falsely negative results. This suggests Cdc42 use in many more viral species is deserving of reinvestigation. Many of the studies mentioned in this review did not employ Cdc42-specific agents unfortunately, leaving the literature unable to equally characterise Cdc42 use amongst the viral species mentioned, signposting a clear need for further research into even the fundamentals of this topic.

Furthermore, though the renowned diversity of RNA virus species gives rise to ample inter-species variation even within this narrow realm, this review identified two specific scenarios of Cdc42-signalling induction in cell entry processes that unite the divergent species discussed here. Firstly, entry via induction of macropinocytosis, which seems to involve obligatory Cdc42 use, is increasingly being recognised as an alternative, and sometimes preferred, method of entry that is shared between many viruses, including the EBOV and RSV discussed earlier. Determining whether or not the induction of Cdc42-signalling in these macropinocytotic entry processes differs between these species could identify conserved viral processes, and should be researched further.

Secondly, one particular Cdc42 pathway can be identified as uniting multiple RNA viruses in the studies mentioned here: the Cdc42-WASP-Arp2/3 pathway promoted by intersectin. Inhibition of constituents, and presumed specific targeting of the entire pathway via ZCL278, has been shown to inhibit infectivity in HIV-1, CoV and RSV, suggesting a conserved component in entry processes between these divergent species. Considering that within the field of HIV-1 alone there have been calls for the consideration of, and even one attempt of employing, GTPase-inhibiting agents in treatment [33,68], it is tempting to speculate that pharmacological targeting of viral Cdc42-WASP-Arp2/3 use could constitute the basis of broad-spectrum anti-viral therapy beyond just HIV-1. Presently, however, we remain hamstringed by our limited knowledge of the pathways, molecules, and even agents of inhibition involved. Further investigation of viral use of the Cdc42-WASP-Arp2/3 pathway is clearly warranted, studying specific agents such as ZCL278 in greater detail.

## References

[1] Pollard T.D., Cooper J.A. (2009). Actin, a central player in cell shape and movement. Science.

[2] Blanchoin L., Boujemaa-Paterski R., Sykes C., Plastino J. (2014). Actin dynamics, architecture, and mechanics in cell motility. Physiol. Rev..

[3] Pantaloni D., Le Clainche C., Carlier M.F. (2001). Mechanism of actin-based motility. Science.

[4] Delorme-Axford E., Coyne C.B. (2011). The Actin Cytoskeleton as a Barrier to Virus Infection of Polarized Epithelial Cells. Viruses.

[5] Ridley A.J. (2006). Rho GTPases and actin dynamics in membrane protrusions and vesicle trafficking. Trends Cell Biol..

[6] Kurokawa K., Itoh R.E., Yoshizaki H., Nakamura Y.O.T., Matsuda M. (2004). Coactivation of Rac1 and Cdc42 at Lamellipodia and Membrane Ruffles Induced by Epidermal Growth Factor. Mol. Biol. Cell.

[7] Diebold B.A., Fowler B., Lu J., Dinauer M.C., Bokoch G.M. (2004). Antagonistic cross-talk between Rac and Cdc42 GTPases regulates generation of reactive oxygen species. J. Biol. Chem..

[8] Guilluy C., Garcia-Mata R., Burridge K. (2011). Rho protein crosstalk: Another social network?. Trends Cell Biol..

[9] Holzinger A. (2001). Jasplakinolide: An actin-specific reagent that promotes actin polymerization. Methods Mol. Biol..

[10] Voth D.E., Ballard J.D. (2005). Clostridium difficile Toxins: Mechanism of Action and Role in Disease. Clin. Microbiol. Rev..

[11] Orgaz J.L., Herraiz C., Sanz-Moreno V. (2014). Rho GTPases modulate malignant transformation of tumor cells. Small GTPases.

[12] Arias-Romero L.E., Chernoff J. (2013). Targeting Cdc42 in cancer. Expert Opin. Ther. Targets.

[13] Lane J., Martin T., Weeks H.P., Jiang W.G. (2014). Structure and role of WASP and WAVE in Rho GTPase signalling in cancer. Cancer Genom. Proteom..

[14] Wilson K.F., Erickson J.W., Antonyak M.A., Cerione R.A. (2013). Rho GTPases and their roles in cancer metabolism. Trends Mol. Med..

[15] Samstag Y., Eibert S.M., Klemke M., Wabnitz G.H. (2003). Actin cytoskeletal dynamics in T lymphocyte activation and migration. J. Leukoc. Biol..

[16] Baldauf H.-M., Pan X., Erikson E., Schmidt S., Daddacha W., Burggraf M., Schenkova K., Ambiel I., Wabnitz G., Gramberg T. (2012). SAMHD1 restricts HIV-1 infection in resting CD4(+) T cells. Nat. Med..

[17] Zack J.A., Arrigo S.J., Weitsman S.R., Go A.S., Haislip A., Chen I.S.Y. (2015). HIV-1 entry into quiescent primary lymphocytes: Molecular analysis reveals a labile, latent viral structure. Cell.

[18] Vatakis D.N., Nixon C.C., Zack J.A. (2010). Quiescent T cells and HIV: An unresolved relationship. Immunol. Res..

[19] Ganesh L., Burstein E., Guha-Niyogi A., Louder M.K., Mascola J.R., Klomp L.W.J., Wijmenga C., Duckett C.S., Nabel G.J. (2003). The gene product Murr1 restricts HIV-1 replication in resting CD4+ lymphocytes. Nature.

[20] Pan X., Baldauf H.-M., Keppler O.T., Fackler O.T. (2013). Restrictions to HIV-1 replication in resting CD4(+) T lymphocytes. Cell Res..

[21] Lassen K., Han Y., Zhou Y., Siliciano J., Siliciano R.F. (2004). The multifactorial nature of HIV-1 latency. Trends Mol. Med..

[22] Han Y., Wind-Rotolo M., Yang H.-C., Siliciano J.D., Siliciano R.F. (2007). Experimental approaches to the study of HIV-1 latency. Nat. Rev. Microbiol..

[23] Yu D., Wang W., Yoder A., Spear M., Wu Y. (2009). The HIV envelope but not VSV glycoprotein is capable of mediating HIV latent infection of resting CD4 T cells. PLoS Pathog..

[24] Cicala C., Arthos J., Selig S.M., Dennis G.J., Hosack D.A., Van Ryk D., Spangler M.L., Steenbeke T.D., Khazanie P., Gupta N. (2002). HIV envelope induces a cascade of cell signals in non-proliferating target cells that favor virus replication. Proc. Natl. Acad. Sci. USA.

[25] Jimenez-Baranda S., Gomez-Mouton C., Rojas A., Martinez-Prats L., Mira E., Ana Lacalle R., Valencia A., Dimitrov D.S., Viola A., Delgado R. (2007). Filamin-A regulates actin-dependent clustering of HIV receptors. Nat. Cell Biol..

[26] Wu Y., Yoder A., Yu D., Wang W., Liu J., Barrett T., Wheeler D., Schlauch K. (2008). Cofilin activation in peripheral CD4 T cells of HIV-1 infected patients: A pilot study. Retrovirology.

[27] Yoder A., Yu D., Dong L., Iyer S.R., Xu X., Kelly J., Liu J., Wang W., Vorster P.J., Agulto L. (2008). HIV envelope-CXCR4 signaling activates cofilin to overcome cortical actin restriction in resting CD4 T cells. Cell.

[28] Barrero-Villar M., Cabrero J.R., Gordon-Alonso M., Barroso-Gonzalez J., Alvarez-Losada S., Munoz-Fernandez M.A., Sanchez-Madrid F., Valenzuela-Fernandez A. (2009). Moesin is required for HIV-1-induced CD4-CXCR4 interaction, F-actin redistribution, membrane fusion and viral infection in lymphocytes. J. Cell Sci..

[29] Vorster P.J., Guo J., Yoder A., Wang W., Zheng Y., Xu X., Yu D., Spear M., Wu Y. (2011). LIM kinase 1 modulates cortical actin and CXCR4 cycling and is activated by HIV-1 to initiate viral infection. J. Biol. Chem..

[30] Spear M., Guo J., Wu Y. (2012). The trinity of the cortical actin in the initiation of HIV-1 infection. Retrovirology.

[31] Spear M., Guo J., Turner A., Yu D., Wang W., Meltzer B., He S., Hu X., Shang H., Kuhn J. (2014). HIV-1 triggers WAVE2 phosphorylation in primary CD4 T cells and macrophages, mediating Arp2/3-dependent nuclear migration. J. Biol. Chem..

[32] Pontow S., Harmon B., Campbell N., Ratner L. (2007). Antiviral activity of a Rac GEF inhibitor characterized with a sensitive HIV/SIV fusion assay. Virology.

[33] Denton P.W., Othieno F., Martinez-Torres F., Zou W., Krisko J.F., Fleming E., Zein S., Powell D.A., Wahl A., Kwak Y.T. (2011). One percent tenofovir applied topically to humanized BLT mice and used according to the CAPRISA 004 experimental design demonstrates partial protection from vaginal HIV infection, validating the BLT model for evaluation of new microbicide candidates. J. Virol..

[34] Amy C., Dittmar M.T. (2013). The Role of GTPases and Their Regulators within Early Steps of the HIV-1 Lifecycle, and Their Potential as a Target against HIV-1 Infection.

[35] Swaine T., Dittmar M.T. (2014). The Effect of Pharmacological Inhibition of RhoA and Cdc42 on the Early Steps of the HIV-1 Infection Lifecycle.

[36] Chen C., Song X., Ma S., Wang X., Xu J., Zhang H., Wu Q., Zhao K., Cao J., Qiao J. (2015). Cdc42 inhibitor ML141 enhances G-CSF-induced hematopoietic stem and progenitor cell mobilization. Int. J. Hematol..

[37] Surviladze Z., Waller A., Strouse J.J., Bologa C., Ursu O., Salas V., Parkinson J.F., Phillips G.K., Romero E., Wandinger-Ness A. (2010). A Potent and Selective Inhibitor of Cdc42 GTPase. Probe Reports from the NIH Molecular Libraries Program [Internet].

[38] Chen H.-Y., Yang Y.M., Stevens B.M., Noble M. (2013). Inhibition of redox/Fyn/c-Cbl pathway function by Cdc42 controls tumour initiation capacity and tamoxifen sensitivity in basal-like breast cancer cells. EMBO Mol. Med..

[39] Friesland A., Zhao Y., Chen Y.-H., Wang L., Zhou H., Lu Q. (2013). Small molecule targeting Cdc42-intersectin interaction disrupts Golgi organization and suppresses cell motility. Proc. Natl. Acad. Sci. USA.

[40] Malinowsky K., Luksza J., Dittmar M.T. (2008). Susceptibility to virus-cell fusion at the plasma membrane is reduced through expression of HIV gp41 cytoplasmic domains. Virology.

[41] Lohrengel S., Hermann F., Hagmann I., Oberwinkler H., Scrivano L., Hoffmann C., von Laer D., Dittmar M.T. (2005). Determinants of human immunodeficiency virus type 1 resistance to membrane-anchored gp41-derived peptides. J. Virol..

[42] Hong L., Kenney S.R., Phillips G.K., Simpson D., Schroeder C.E., Noth J., Romero E., Swanson S., Waller A., Strouse J.J. (2013). Characterization of a Cdc42 protein inhibitor and its use as a molecular probe. J. Biol. Chem..

[43] Hussain N.K., Jenna S., Glogauer M., Quinn C.C., Wasiak S., Guipponi M., Antonarakis S.E., Kay B.K., Stossel T.P., Lamarche-Vane N. (2001). Endocytic protein intersectin-l regulates actin assembly via Cdc42 and N-WASP. Nat. Cell Biol..

[44] Jenna S., Hussain N.K., Danek E.I., Triki I., Wasiak S., McPherson P.S., Lamarche-Vane N. (2002). The activity of the GTPase-activating protein CdGAP is regulated by the endocytic protein intersectin. J. Biol. Chem..

[45] Klein I.K., Predescu D.N., Sharma T., Knezevic I., Malik A.B., Predescu S. (2009). Intersectin-2L regulates caveola endocytosis secondary to Cdc42-mediated actin polymerization. J. Biol. Chem..

[46] McGavin M.K., Badour K., Hardy L.A., Kubiseski T.J., Zhang J., Siminovitch K.A. (2001). The intersectin 2 adaptor links Wiskott Aldrich Syndrome protein (WASp)-mediated actin polymerization to T cell antigen receptor endocytosis. J. Exp. Med..

[47] Srinivasakumar N., Ogra P.L., Flanagan T.D. (1991). Characteristics of fusion of respiratory syncytial virus with HEp-2 cells as measured by R18 fluorescence dequenching assay. J. Virol..

[48] Ohki S., Liu J.-Z., Schaller J., Welliver R.C. (2003). The compound DATEM inhibits respiratory syncytial virus fusion activity with epithelial cells. Antivir. Res..

[49] Razinkov V., Huntley C., Ellestad G., Krishnamurthy G. (2002). RSV entry inhibitors block F-protein mediated fusion with model membranes. Antivir. Res..

[50] Huang K., Incognito L., Cheng X., Ulbrandt N.D., Wu H. (2010). Respiratory Syncytial Virus-Neutralizing Monoclonal Antibodies Motavizumab and Palivizumab Inhibit Fusion. J. Virol..

[51] Kolokoltsov A.A., Deniger D., Fleming E.H., Roberts N.J., Karpilow J.M., Davey R.A. (2007). Small Interfering RNA Profiling Reveals Key Role of Clathrin-Mediated Endocytosis and Early Endosome Formation for Infection by Respiratory Syncytial Virus. J. Virol..

[52] San-Juan-Vergara H., Sampayo-Escobar V., Reyes N., Cha B., Pacheco-Lugo L., Wong T., Peeples M.E., Collins P.L., Castano M.E., Mohapatra S.S. (2012). Cholesterol-rich microdomains as docking platforms for respiratory syncytial virus in normal human bronchial epithelial cells. J. Virol..

[53] Krzyzaniak M.A., Zumstein M.T., Gerez J.A., Picotti P., Helenius A. (2013). Host Cell Entry of Respiratory Syncytial Virus Involves Macropinocytosis Followed by Proteolytic Activation of the F Protein. PLoS Pathog..

[54] Peterson J.R., Lebensohn A.M., Pelish H.E., Kirschner M.W. (2006). Biochemical suppression of small molecule inhibitors: A new strategy to identify inhibitor targets and signaling pathway components. Chem. Biol..

[55] Cuadras M.A., Arias C.F., Lopez S. (1997). Rotaviruses induce an early membrane permeabilization of MA104 cells and do not require a low intracellular Ca^2+^ concentration to initiate their replication cycle. J. Virol..

[56] Bass D.M., Baylor M., Chen C., Upadhyayula U. (1995). Dansylcadaverine and cytochalasin D enhance rotavirus infection of murine L cells. Virology.

[57] Kaljot K.T., Shaw R.D., Rubin D.H., Greenberg H.B. (1988). Infectious rotavirus enters cells by direct cell membrane penetration, not by endocytosis. J. Virol..

[58] Sánchez-San Martín C., López T., Arias C.F., López S. (2004). Characterization of Rotavirus Cell Entry. J. Virol..

[59] Díaz-Salinas M.A., Romero P., Espinosa R., Hoshino Y., López S., Arias C.F. (2013). The Spike Protein VP4 Defines the Endocytic Pathway Used by Rotavirus To Enter MA104 Cells. J. Virol..

[60] Gutiérrez M., Isa P., Sánchez-San Martín C., Pérez-Vargas J., Espinosa R., Arias C.F., López S. (2010). Different Rotavirus Strains Enter MA104 Cells through Different Endocytic Pathways: The Role of Clathrin-Mediated Endocytosis. J. Virol..

[61] Díaz-Salinas M.A., Silva-Ayala D., López S., Arias C.F. (2014). Rotaviruses Reach Late Endosomes and Require the Cation-Dependent Mannose-6-Phosphate Receptor and the Activity of Cathepsin Proteases To Enter the Cell. J. Virol..

[62] Wang H., Yang P., Liu K., Guo F., Zhang Y., Zhang G., Jiang C. (2008). SARS coronavirus entry into host cells through a novel clathrin- and caveolae-independent endocytic pathway. Cell Res..

[63] Nomura R., Kiyota A., Suzaki E., Kataoka K., Ohe Y., Miyamoto K., Senda T., Fujimoto T. (2004). Human Coronavirus 229E Binds to CD13 in Rafts and Enters the Cell through Caveolae. J. Virol..

[64] Koivusalo M., Welch C., Hayashi H., Scott C.C., Kim M., Alexander T., Touret N., Hahn K.M., Grinstein S. (2010). Amiloride inhibits macropinocytosis by lowering submembranous pH and preventing Rac1 and Cdc42 signaling. J. Cell Biol..

[65] Aleksandrowicz P., Marzi A., Biedenkopf N., Beimforde N., Becker S., Hoenen T., Feldmann H., Schnittler H.-J. (2011). Ebola Virus Enters Host Cells by Macropinocytosis and Clathrin-Mediated Endocytosis. J. Infect. Dis..

[66] Quinn K., Brindley M.A., Weller M.L., Kaludov N., Kondratowicz A., Hunt C.L., Sinn P.L., McCray P.B., Stein C.S., Davidson B.L. (2009). Rho GTPases Modulate Entry of Ebola Virus and Vesicular Stomatitis Virus Pseudotyped Vectors. J. Virol..

[67] Mulherkar N., Raaben M., de la Torre J.C., Whelan S.P., Chandran K. (2011). The Ebola virus glycoprotein mediates entry via a non-classical dynamin-dependent macropinocytic pathway. Virology.

[68] Spear M., Guo J., Wu Y. (2013). Novel anti-HIV therapeutics targeting chemokine receptors and actin regulatory pathways. Immunol. Rev..

